# A Novel qPCR Method for the Detection of Lactic Acid Bacteria in Fermented Milk

**DOI:** 10.3390/foods10123066

**Published:** 2021-12-09

**Authors:** Xiankang Fan, Xiefei Li, Tao Zhang, Jue Xu, Zihang Shi, Zhen Wu, Jihuan Wu, Daodong Pan, Lihui Du

**Affiliations:** 1 State Key Laboratory for Managing Biotic and Chemical Threats to the Quality and Safety of Agro-Products, Ningbo University, Ningbo 315211, China; fanxiankang@foxmail.com (X.F.); xiefeili.edu@gmail.com (X.L.); tao_zhang2021@163.com (T.Z.); joy123xj@163.com (J.X.); szh11866@163.com (Z.S.); woodsen@163.com (Z.W.); dulihui@nbu.edu.cn (L.D.); 2 Key Laboratory of Animal Protein Food Processing Technology of Zhejiang Province, College of Food and Pharmaceutical Sciences, Ningbo University, Ningbo 315832, China; 3 Ningbo Yifule Biotechnology Co., Ltd., Ningbo 315500, China; wujihuan@jellebrown.com

**Keywords:** qPCR, lactic acid bacteria, yogurt, laser confocal microscope, *tuf* gene, flow cytometry

## Abstract

The number of live lactic acid bacteria (LAB) is an important quality indicator for yogurt, the quantitative testing of LAB has become an important task in the evaluation of product quality and function. By analyzing and comparing the performance of 16S rRNA gene and *tuf* gene used in absolute quantification, the *tuf* gene with copy number 1 was selected as the target gene of six LAB. By drawing a standard curve to achieve qualitative and quantitative detection of six strains of LAB, the detection range was found to be 1 × 10^3^–1 × 10^8^ copies/µL. The traditional plate colony count and Flow Cytometry (FCM) were compared with the method of qPCR, which was used in this experiment. Meanwhile, the confocal laser microscope combined with STYO 9 and propidium iodide dyes was used to determine that the content of viable bacteria in the yogurt was more than 90%, which proved that the detection result using qPCR method was closer to the true level of LAB in yogurt. Compared with the existing methods, the method in this study allowed the qualitative and quantitative detection of the six kinds of LAB in yogurt, and the distribution of live and dead bacteria in yogurt could be calculated.

## 1. Introduction

Lactic acid bacteria (LAB) starter is widely used in the production of dairy products because of its probiotic effect [1]. Yogurt is one of the best sources of LAB for the human body. It is made by co-fermentation of *Lactobacillus delbrueckii* subsp. *bulgaricus* and *Streptococcus thermophilus* that has functions, such as anti-colon cancer [2], diarrhea inhibition [3], prevention of intestinal inflammation to relieve depression [4], and degradation of ciprofloxacin [5]. Therefore, the number of LAB is an important indicator of yogurt quality, and the Chinese national standard stipulates that the number of LAB in yogurt shall not be less than 10^6^ CFU/mL [6]. 

Quantitative detection of LAB has become an important task for product quality and function evaluation [7]. At present, the traditional plate counting method is generally used to determine the number of LAB in yogurt. However, due to the complexity of yogurt strains, it was difficult to determine the content of each LAB in yogurt through the traditional plate colony counting method. This method has large operating errors, is time-consuming, and has low sensitivity. Moreover, it cannot accurately and quickly reflect the dynamic changes of the flora during the fermentation process [8]. The selective medium used was not able to count the bacterial cells that were difficult to cultivate although they had metabolic activity, and its selectivity was weak for yogurt samples containing a variety of LAB [9]. Meanwhile, the use of Flow Cytometry (FCM) as another method to enumerate LAB was also reported by Salma et al. who used a combination of FCM and BOX/PI dyes to determine LAB in wine [10]. The FCM method can identify the ratio of live/dead bacteria for separate enumeration, but it cannot specifically target one or more strains of bacteria in the sample [11]. In recent years, researchers have developed molecular biology methods to detect LAB, and the most widely used method was qPCR. The qPCR method is a method to quantify the initial template in the sample to be tested, including SYBR Green dye and TaqMan fluorescent probe, by labeling the PCR products with fluorescent dyes or fluorescently labeled specific probes and analyzing the results with the instrument and corresponding software. This method has a fast detection speed and high sensitivity compared to traditional PCR, and it is also non-polluting, strongly specific, reproducible, and has a short detection time. Therefore, it has been applied for the detection of dominant and harmful bacteria in food. Quijada et al. used the PMA-qPCR (Combination of propidium monoazide and quantitative polymerase chain reaction technology) method to detect human adenovirus-2 and portal viruses in Spanish fermented sausages, and found that this method can quickly detect viruses that cannot be cultivated under the current technology [12]. For the first time, Nordstrom used multiplex real-time quantitative PCR to detect *Salmonella* in chicken [13]. Determining the amount of each type of LAB in fermented yogurt was our initial aim, but the existing methods have some problems such as not being able to specifically distinguish the amount of each type of LAB, complicated detection methods, long time required for detection, and not being able to distinguish live bacteria from dead bacteria. Therefore, the objective of this study was to establish a method based on qPCR that could rapidly detect qualitatively and quantitatively six kinds of LAB in yogurt and could distinguish the distribution of six kinds of LAB between live and dead bacteria in yogurt.

The advantages of this study were as follows. Firstly, we chose the *tuf* gene with copy number of 1 instead of the 16S rRNA gene, whose copy number varies with the strain. Second, this assay could qualitatively and quantitatively quantify six species of LAB simultaneously. Third, the laser confocal results showed that the assay results are closer to the real values. The technology could be used not only for rapid detection of LAB in yogurt but also for the detection of contaminating bacteria in fermented food.

## 2. Materials and Methods

### 2.1. Material

The 6 kinds of LAB used in this experiment were Lactobacillus delbrueckii subsp. bulgaricus CICC 6045 (LD), Streptococcus thermophilus CGMCC1.1864 (ST), Lactobacillus acidophilus CICC6074 (LA), Limosilactobacillus fermentum CGMCC1.7434 (LF), Levilactobacillus brevis CGMCC1.5954 (LB), and Lacticaseibacillus casei CGMCC1.5956 (LC). Strains were purchased from China General Microbiological Culture Collection Center (CGMCC) and deposited in the laboratory of Ningbo University. Plasmid pUC 19 and LIVE/DEAD BacLight Bacterial Viability Kit (containing SYTO9 and PI) were purchased from Takara and Thermo Fisher Scientific, respectively. E.Z.N.A. Bacterial DNA Kit and E.Z.N.A. Gel Extraction kit were produced by Omega, USA. Proteinase K and sodium dodecyl sulfate (SDS) were purchased from Biotech Biological Engineering Co (Shanghai, China). 

### 2.2. Extraction of DNA from LAB and Compared 16S rRNA Gene with Tuf Gene

Bacterial DNA extraction kit was used to extract the DNA of 6 strains of LAB and determine their concentration and purity. To extract LAB from yogurt, the yogurt was diluted 15 times and then mixed with proteinase K and 8.0% (*w/v*) sodium dodecyl sulfate (SDS), and finally extracted with the above kit. The extracted DNA was stored in the refrigerator at −20 °C. The rrnDB database was used to compare the copy number of 16S rRNA gene and *tuf* gene of 6 strains of LAB [14].

### 2.3. Design and Synthesis of Primers

The primer design software Primer Premier 6 was used to design specific primers for 6 strains of LAB, and the length of the amplified fragments of the primers was about 150 bp. The specificity of the primers was evaluated by using the Basic Local Alignment Search Tool (BLAST) database on the NCBI website [15]. The software CE Design was used to develop cloning primers with homology arms. Afterward, the designed primers were synthesized by Shenggong Biological Engineering Co., Ltd. (Shanghai, China), Then, the LAB DNA of known species isolated were used to experimentally verify the specificity of the designed primers, as follows: *Lactobacillus delbrueckii* subsp. *bulgaricus CICC 6045* (LB), *Streptococcus thermophilus CGMCC1.1864* (ST), *Lactobacillus acidophilus CICC6074* (LA), *Limosilactobacillus fermentum CGMCC1.7434* (LF), *Levilactobacillus brevis CGMCC1.5954* (LB), *Lacticaseibacillus casei CGMCC1.5956* (LC). In the 6 reaction system groups, 7 tubes were set in parallel. In reaction systems 1–6, the primers were LA, LB, LC, LD, LF, and ST. The templates in each reaction system were the extracted DNA of 6 strains, and 1 tube of negative control. PCR reaction conditions and procedures are shown in Table 1 and Table 2. The PCR reaction product was checked by 1% (*w*/*v*) agarose gel electrophoresis, the electrophoresis condition was 130 V constant pressure for 30 min, and the test result was analyzed by the gel imaging system.

### 2.4. Plasmid Extraction and Restriction Digestion

In order to construct a plasmid carrying the target gene that could be used to plot a standard curve by qPCR, plasmid cloning needed to be performed. The plasmid pUC19 was extracted from *E. scherichia coli*, and PCR was used to verify the extraction results. Afterward, SacI and Hindlll were chosen as the double restriction sites, and the restriction reaction system is shown in Table 3. After adding the sample, the mixture was repeatedly pipetted and mixed vigorously. It was kept in a metal bath at 37 °C for 10 min, and transferred to 80 °C for inactivation for 15 min. Finally, the digestion results were observed by gel electrophoresis. Primer 2 was used to perform PCR amplification and tapping recovery of the target gene, and the amplification procedure was shown in Table 1 and Table 2. The PCR reaction product was subjected to gel electrophoresis. Afterward, fully separated DNA fragments were cut out, and the DNA was finally recovered using a DNA tapping recovery kit.

### 2.5. TA Cloning of the Target Gene

LB solid medium, which contained 100 µg/mL ampicillin (Amp) solution were prepared for 300 mL. The ligation reaction system (10 μL) comprised the following: 1 μL of purified PCR product, 1 μL of pUC19 linearized vector, 5 μL of 2× ClonExpress Mix, and 3 μL of ddH_2_O. The sample was pipetted gently for mixing, connected at 50 °C for 5 min, and reduced to 4 °C [16,17]. The ligation product was added to 100 μL of DH5α competent cells to transform the recombinant plasmid, which was placed on ice for 30 min, 42 °C for 45 s, and immediately cooled on ice for 2–3 min. After the 900 μL of LB liquid medium was added, the mixture was at 37 °C, shaken at 200–250 rpm for 1 h. The recovered bacteria were aspirated and spread on an agar medium containing ampicillin (Amp), inverted the culture at 37 °C for 12–16 h, picked a single positive colony from the plate for PCR detection, and sent it to Shenggong Biotechnology Co., Ltd. (Shanghai, China), for sequencing.

### 2.6. Build qPCR Standard Curve

Positive strains were picked and inoculated into Amp-containing LB liquid medium. They were placed at 37 °C and 200 r·min^−1^ overnight, and plasmids were extracted. The concentration of the standard plasmid was determined and the copy number concentration in the stock solution of the standard plasmid was calculated. After performing a 10-fold serial dilution, qPCR was performed. A standard curve was drawn with the threshold cycle number Cq as the ordinate and the logarithm of the concentration of the reference substance as the abscissa. Fluorescence quantitative reaction system (20 μL) comprised the following: 3 μL of ddH_2_O, 2 μL of PCR Primer, 10 μL of Master Mix 2× and 5 μL of DNA.

### 2.7. Determine the Content of Each Strain in the Fermentation Broth and Yogurt

Single colonies of 6 strains were picked and put into MRS broth, which were cultured at 37 °C for 12 h. After 10-fold serial dilution, the bacterial solution was centrifuged at 8000× *g* and 4 °C for 5 min, and the DNA of the target strain was extracted for qPCR. At the same time, the plate colony was counted for comparison.

Tetra Pak whole milk was used; 7% sucrose was added; and the mixture was homogenized at 65 °C for 30 min, sterilized at 95 °C for 10 min, and then cooled at 42 °C for 20 min. The strain combination for LAB-fermented yoghurt were as follows: ST + LD; ST + LD + LB; ST + LD + LA; ST + LD + LF; and ST + LD + LC. An additional group of blank milk without LAB inoculation was set up as the negative group. The number of each pure-species LAB was controlled to be 5 × 10^6^ CFU/mL, and the cells were collected by 8000× *g*, at 4 °C and centrifuged for 5 min. After washing twice with sterile normal saline (0.85%, *w/v*), the bacteria were resuspended in the reconstituted milk and fermented at 42 °C for 6 h. The fermented yogurt was diluted in a 10-fold series, 10,000× *g* and centrifuged at 4 °C for 5 min. Then the bacteria were collected. Afterward, the DNA of all LAB in the yogurt were extracted by using Bacterial DNA Kit, which was utilized as a template for qPCR reactions to perform qPCR and compared with the standard curve.

After the yogurt was homogenized, it was diluted 10^9^ times with sterile saline and incubated on M17 medium at 37 °C for 24 h to count *Streptococcus thermophilus* in the yogurt [16]. For the enumeration of *Limosilactobacillus fermentum* in yogurt, the MRS medium was used, and the coated plates were inverted and incubated in an anaerobic incubator at 36 ± 1 °C for 72 ± 2 h. *Levilactobacillus brevis* was counted after incubation on MRS medium at 30 °C under aerobic conditions for 36 h. The *Lacticaseibacillus casei* in yogurt was counted by adding 50 mg/L vancomycin after 48 h of anaerobic incubation at 37 °C and then incubated for 72 h at 37 °C in an anaerobic environment. We calculated the content of *Lactobacillus acidophilus* in the yogurt by the anaerobic culture of MRS maltose agar plates at 37 °C from 48 h to 72 h. The method for the enumeration of *Lactobacillus delbrueckii subsp. bulgaricus* in each type of sample was shown below. (ST-LD): the pH of MRS medium to 5.7 was adjusted and then incubated at 37 °C anaerobically for 48 h; (LA-LD): we incubated the MRS medium at pH 4.58 in anaerobic incubator at 43 °C for 72 h; (LF-LD): we used MRS medium at 37 °C anaerobically for 48 h; (LC-LD): diluted yogurt or solution was coated on MRS plates at pH 5.2 and placed under anaerobic incubation conditions at 43 °C for 72 h; (LB-LD): anaerobic incubation was performed on MRS medium at 37 °C for 5 days [18]. All steps were performed in three parallels.

### 2.8. Enumeration of LAB Using FCM

The fermented yogurt samples were diluted 100 times using sterile PBS, homogenized and passed through a 48 μm sterile filter membrane. Then, 3 μL of SYTO 9 and propidium iodide (PI) dye were mixed with 1 mL of the sample, which was then incubated for 15 min away from light and then passed through the membrane again. Detection was performed using Analytical Flow Cytometer (Beckman Coulter, Inc., Brea, CA, USA). The FL1 detection channel was selected according to the maximum excitation wavelength of 483 nm and the maximum emission wavelength of 503 nm was selected for the green fluorescent dye in SYTO9 single dyeing. Similarly, the FL3 detection channel was selected for the PI red fluorescent dye. The number of cells of bacteria in a 1 m L suspension can be calculated by the following equation:

Ns = (Vs × Nd)/(vd × td)(1)

where Ns (cells/mL) is the number of cells in suspension; Vs. (mL) is the volume of the suspension; νd (μL/min) is the flow rate set in the flow cytometer 30 μL/min is the medium speed; Nd (cells/mL) is the number of cells detected; and td (s) is the detection time.

### 2.9. SYTO 9 Combined with PI Stains to Distinguish Live and Dead Bacteria

The fermented yogurt developed in step 2.7 was diluted 10^−3^ times with sterile PBS. At the same time, SYTO 9 and PI dyes in the LIVE/DEAD BacLight Bacterial Viability Kit were added to the microcentrifuge tube in a 1:1 ratio and then mixed repeatedly by pipetting to prepare the dye mixture. Afterward, 3 μL of the dye mixture was added to each milliliter of yogurt diluent, stirred, and mixed well. The mixture was left to stand for 15 min in the dark at room temperature. Stained yogurt diluent (5 μL was collected into the glass slide. The cover glass was installed, and the laser confocal microscope was used to observe the LAB staining, which was performed by selecting the SYTO 9 and PI double staining program.

### 2.10. Statistical Analysis

Experiments were carried out with 3 independent replicates of each sample. Each replicate was analyzed 3 times. Origin 2018 (OriginLab, Northampton, MA, USA) and Spass software (IBM, Armonk, NY, USA) were used to process and analyze the data. The standard deviation was used to calculate the intergroup error, and the curve was fitted by the nonlinear least squares based on the Levernberg–Marquardt algorithm.

## 3. Results

### 3.1. Comparation of 16S rRNA Gene and Tuf Gene

**Figure 1:**
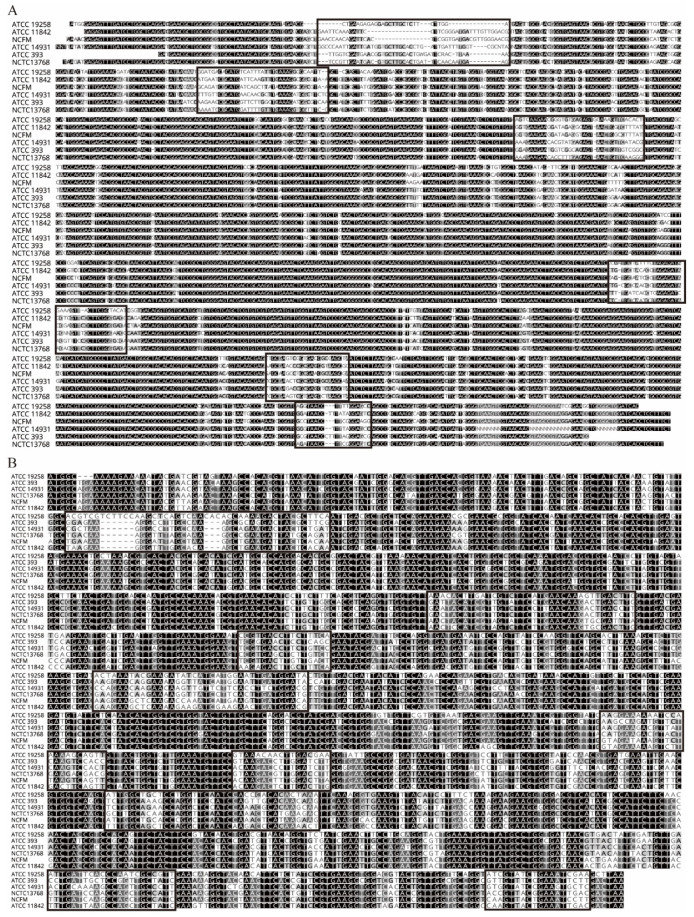
Multiple alignments of 16S rRNA and *tuf* genes of six lactic acid bacteria (LAB) model strains. (**A**): Multiplexed 16S rRNA gene comparison of six model strains of LAB; (**B**): multiple comparison of *tuf* gene of six model strains of LAB.

Table 4 listed the 16S rRNA gene copy numbers of five typical representative strains of six strains of LAB, and it could be found that for the same LAB, the copy numbers were not the same. For example, for *Lactobacillus acidophilus*, there were 8 types of genomes in the database, and the 16S rRNA copy number was between 4 and 5. The median was 4, and the average was 4.1, but the *tuf* gene copy number was only 1. This indicated that the number of *tuf* genes detected was equal to the number of that species of LAB within the sample and was applicable within the same species. In contrast, the 16S rRNA gene had different copy numbers, even between conspecifics, which created a great inconvenience for counting. From the multiple alignment results of the six LAB model strains in Figure 1A,B on the 16S rRNA gene and the *tuf* gene, we observed that the 16S rRNA gene sequence was very conservative and highly similar, had only six variable regions with a total length of 213 bp, the variable regions accounted for 14.2% of the full length of the 16S rRNA gene. By contrast, the *tuf* gene had nine variable regions with a total length of 253 bp, the variable regions accounted for 21.22% of the full length of the *tuf* gene. Therefore, we chose *tuf* gene as the target gene to design primers instead of 16S rRNA gene.

### 3.2. Primer Design and Specificity Verification

**Figure 2:**
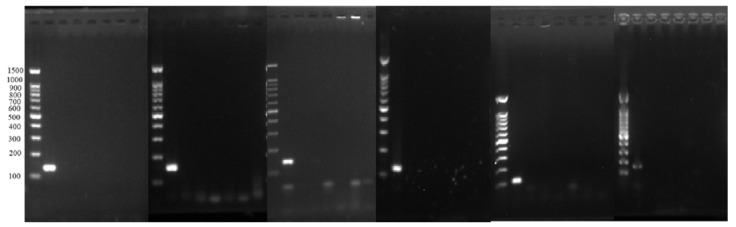
Primer specificity validation. From left to right, the primers in the 6 electropherograms are LA, LB, LC, LD, LF, and ST. The DNA templates in the first well of each electropherogram are LA, LB, LC, LD, LF, and ST, and the last well is a negative control. The DNA templates in the remaining five wells are the remaining five strains of LAB DNA except for the first well.

The primers were compared in the BLAST database to verify the specificity. Table 5 showed the primers designed with specificity, and Table 6 shows the primers carrying the homology arm used to link the target gene to the plasmid. The specificity of these primers was excellent, and the strains commonly used in yogurt could not be amplified. The specificity was verified experimentally and was shown in Figure 2. Only the DNA of the target strain corresponding to the primer was amplified, and the DNA of other strains cannot be amplified.

### 3.3. Reconstruction of Plasmid Vectors

**Figure 3:**
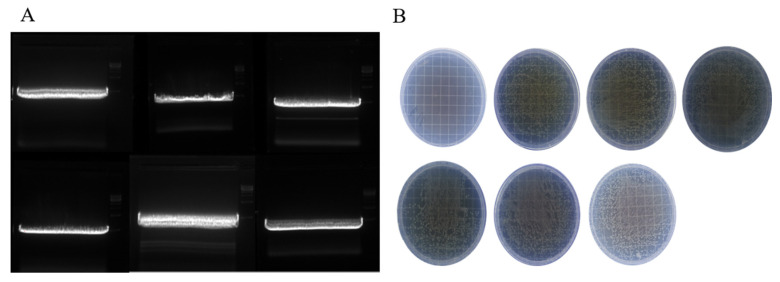
Cut-glue recovery and ligation transformation. (**A**): indicates the results of cut-glue recovery, LA, LB, and LC in the first row in order; and LD, LF, and ST in the second row in order. (**B**): indicates the results of growth on Amp-resistant culture plates after ligation transformation, negative control, LA, LB, LC, LD, LF, and ST in the first row in order.

The aim of this part of the experiment was to modify the pUC19 plasmid by inserting the *tuf* gene of LAB. As shown in Figure 3A, pUC19 was extracted from *E. coli*, which was digested with enzymes, and the bands were sufficiently separated. The recovered bacteria were evenly spread on the agar medium with ampicillin (Amp) resistance. The pUC19 plasmid carried the Amp resistance gene, which, when introduced into *E. coli*, enabled *E. coli* to acquire Amp resistance and grow normally on a plate containing Amp. In Figure 3B, the *E. coli* that was not transformed with pUC19 did not grow on the plate containing ampicillin, and it was transformed with pUC19 (carrying the DNA fragment of the target strain) *E. coli* was grown normally, and the target genes of the six strains were successfully transformed. Positive transformants grown on the plates were expanded, plasmids were extracted and verified by PCR, which confirmed the successful transformation.

### 3.4. Construction of qPCR Standard Curve

**Figure 4:**
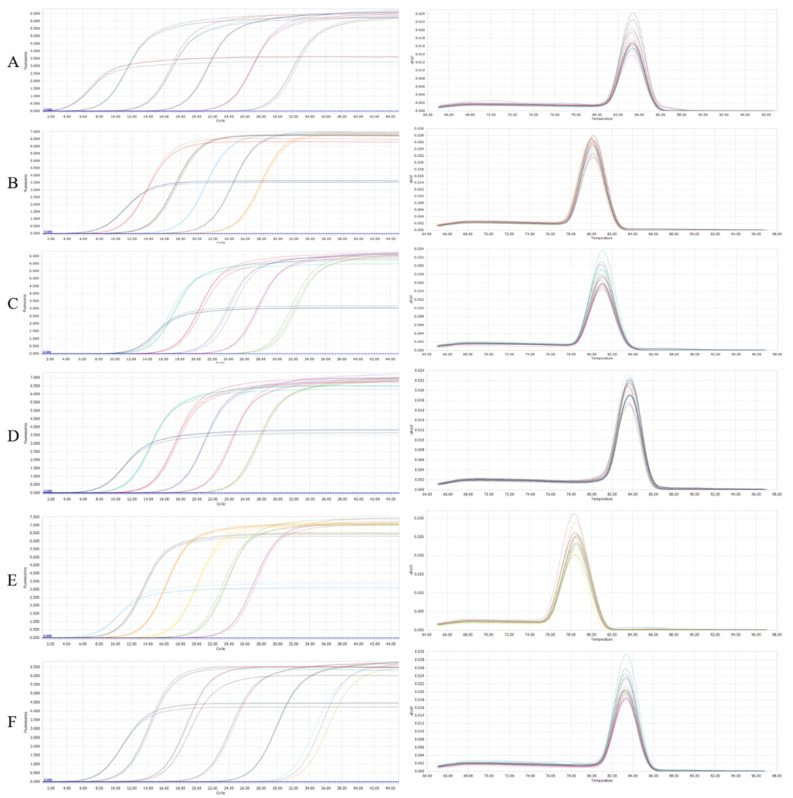
Amplification curve and dissolution curve. The left graph represents the amplification curve and the right graph is the lysis curve. (**A**–**F**) represented *Lactobacillus acidophilus*, *Levilactobacillus brevis*, *Lactobacillus delbrueckii* subsp. *bulgaricus*, *Limosilactobacillus fermentum*, *Streptococcus thermophilus*, and *Lacticaseibacillus casei*, respectively.

As can be seen from the left side of Figure 4, the amplification curve was based on the copy number concentration of the plasmid for gradient amplification. In Figure 4, the melting curves of the six strains were single, with only one peak, indicating the lack of interference from primer dimers and showing a great amplification effect. After the qPCR reaction was completed, the standard curve was plotted using the number of threshold cycles (Cq, the number of cycles taken for the fluorescence signal to reach the set threshold in each reaction tube) as the vertical coordinate and the logarithm of the concentration of the control (pUC19 plasmid) as the horizontal coordinate. The standard curve of six strains was obtained, LA: Y = −3.203X + 34.08 (R^2^ = 0.9977); LB: Y = −3.1011X + 32.72 (R^2^ = 0.9981); LC: Y = −3.2385X + 38.66 (R^2^ = 0.9993); LD: Y = −3.171X + 34.18 (R^2^ = 1.0000); LF: Y = −3.4382X + 35.85 (R^2^ = 0.9972); and ST: Y = −3.1994X + 33.85 (R^2^ = 0.9969). The template showed an excellent linear relationship within the dilution range, and the detection line was 1 × 10^3^–1 × 10^8^ copies/µL.

### 3.5. LAB Content Determination by Traditional Method and qPCR

**Figure 5:**
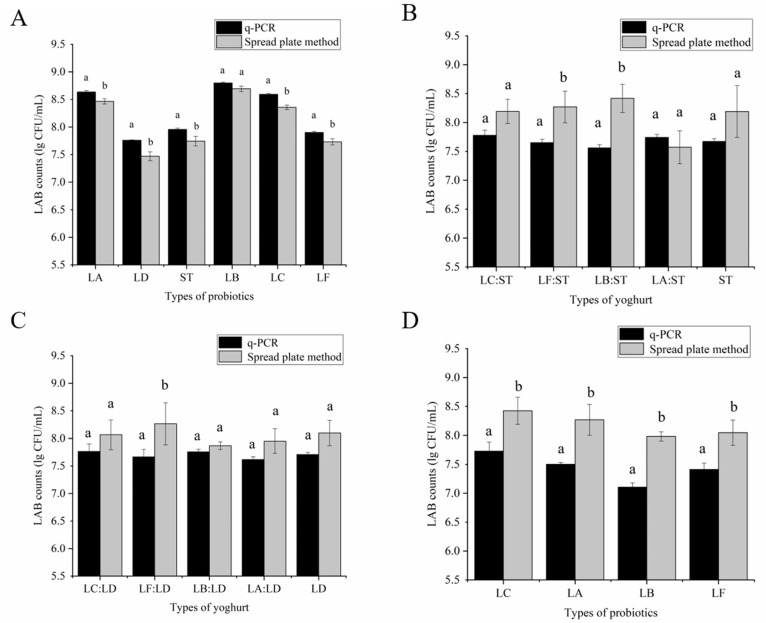
Comparison of spread plate method and qPCR. (**A**): Comparison in the enumeration of six species of LAB monobacterial fermentation. (**B**): LC:ST, LF:ST, LB:ST, LA:ST, and ST; counting ST was represented in yogurt fermented by different combinations of LC + LD + ST, LF + LD + ST, LB + LD + ST, LA + LD + ST, and LD + ST, respectively. (**C**): the LDs in yogurt fermented with different strain combinations were counted. (**D**): indicated that LC, LA, LB, and LF were counted separately in yogurt fermented by different combinations of strains. If *p* < 0.05 indicated a significant difference, *p* ≥ 0.05 indicated that the difference was not significant, the letter “a, b” were used for significance marking.

The number of LAB in the six kinds of single-bacteria fermentation broth were counted on the plate colony by picking the plate with the colony number of 30–300 CFU, and the typical colony of a single pure species appeared on the counting plate. Figure 5A shows that, using the qPCR method gave higher results than the plate colony technique method, and the LA, ST, LD, LC, and LF had significant differences at the *p* < 0.05 level. However, for LB, the two methods were at *p* < 0.05, and the levels of difference were not significant. The results of the two counting methods were similar in the single-bacteria fermentation broth, and the counting result of qPCR method was slightly higher than that of the plate colony.

Figure 5B shows the content of ST, as detected by two methods in five yogurts after 6 h of fermentation. Except for LA:ST, the results of the plate colony counting method were higher than those of the qPCR method. The two groups of LF:ST and LB:ST were significantly different at the *p* < 0.05 level, but the other three groups were not significantly different. For the LD content in yogurt, as shown in Figure 5C, the measurement result of the plate colony counting method was higher than that of the qPCR method. Except for the significant difference in the LF: LD group, no significant difference was found in the other four groups. For the other four kinds of LAB in yogurt, the plate colony count result was significantly higher than the qPCR count result. No LAB or other miscellaneous bacteria were detected by either method in the negative control group that underwent the same treatment without inoculation with LAB. We can conclude that when the six kinds of LAB were counted separately in the mixed fermented yogurt, the plate colony count result was higher than the result obtained using the qPCR method.

### 3.6. Enumeration of LAB Using FCM

**Figure 6:**
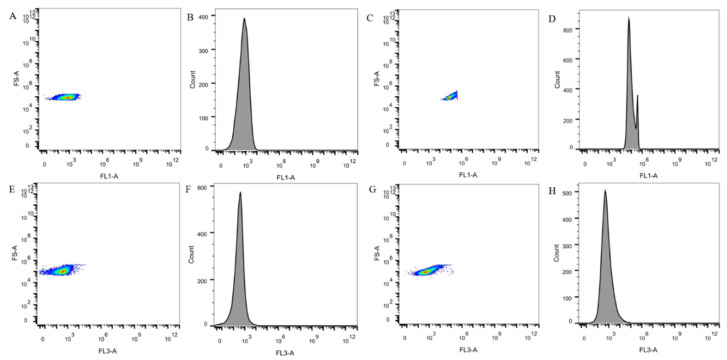
Determination of LAB in Yogurt by Flow Cytometry. (**A**,**E**) were the FCM distribution of SYTO9 and PI single-stained negative control, respectively. (**B**,**F**) were the distribution of SYTO9 and PI single-stained negative control cells and fluorescence intensity, respectively. (**C**,**G**) are the distribution of SYTO9 and PI single-stained positive control flow cells, respectively. (**D**,**H**) are the distribution of SYTO9 and PI single-stained positive control cells and fluorescence intensity, respectively.

Figure 6 showed the results of SYTO 9 with PI single staining, the number of live signals detected in comparison with the blank group was much more than the number of dead signals from the macroscopic view. The numbers of live and dead bacteria in the five yogurts tested using FCM are listed in Table 7. The number of live bacteria ranged from 7.76 lg (CFU/mL) to 7.99 lg (CFU/mL) and the number of dead bacteria ranged from 6.85 to 6.97 lg (CFU/mL). The results were similar to those obtained by using the qPCR method.

### 3.7. Microscopical Examination of Live/Dead Staining

**Figure 7:**
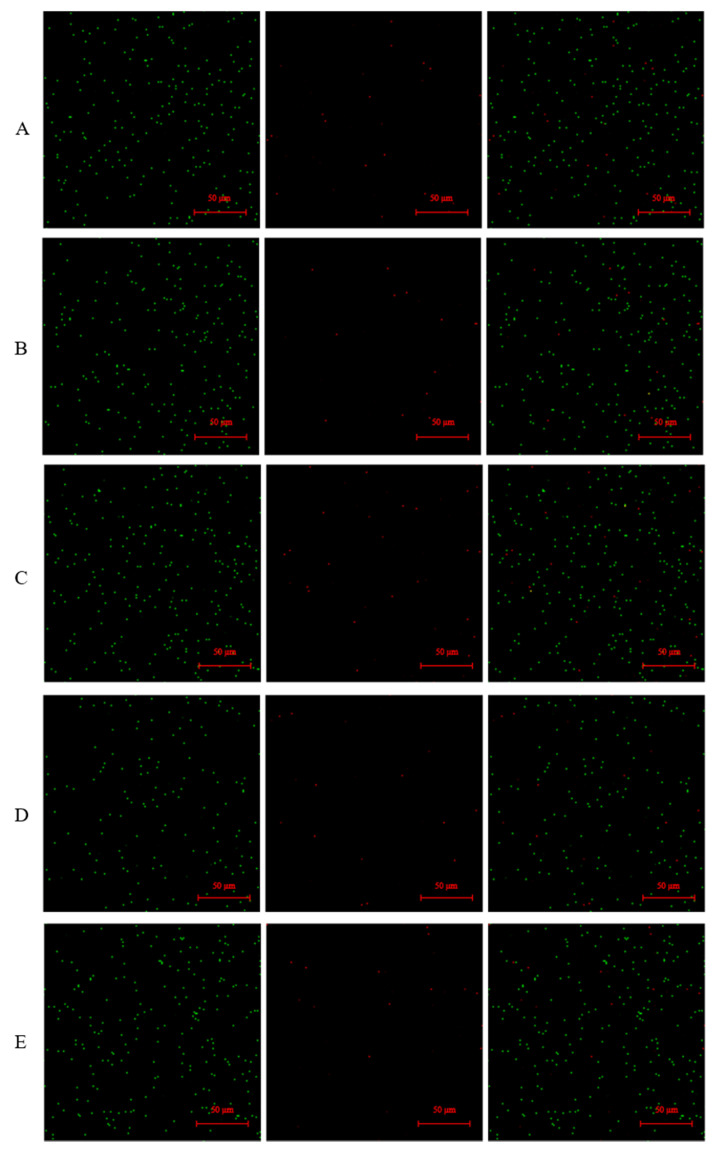
Laser confocal microscopy combined with dye to discriminate between live and dead bacteria. (**A**–**E**) denoted yogurt with different combinations of LC + LD + ST, LF + LD + ST, LB + LD + ST, LA + LD + ST, and LD + ST, respectively. The green fluorescence on the left indicated the live bacteria in the yogurt, whereas the red fluorescence in the middle indicated the dead bacteria in the yogurt, and the combination of the two is shown on the right.

The SYTO 9 was combined with PI stains to be used to distinguish live and dead LAB in the yogurt. Figure 7 was a photo taken by a laser confocal microscope, and we could clearly see that the proportion of green dots in the field of view was very large, but the proportion of red dots was the opposite. After counting the number of green and red spots in the field of view, the proportions of live bacteria could be concluded from Figure 7A–E were 92%, 94.31%, 90.4%, 91.3%, and 93.4%, respectively.

## 4. Discussion

In qPCR reactions, 16S rRNA sequences and some specific gene sequences were typically used as target DNA sequences to design primers or probes [19]. However, the 16S rRNA gene copy number of the same strain was not the same, and the 16S rRNA sequence homology among LAB species was high. Therefore, the selection of the 16S rRNA gene will create errors in our subsequent calculations. By contrast, the *tuf* gene copy number was only 1 [20] and has a highly conservative nature [21]. This indicated that the number of *tuf* genes detected was equal to the number of that species of LAB within the sample and was applicable within the same species. The *tuf* gene was the bacterial extension factor protein, and the coding gene was a housekeeping gene [22]. Other housekeeping gene target sequences were also used, such as Moser A et al. 2017, who used the *pheS* gene as a template sequence to achieve rapid identification and quantification of *Lactobacillus helveticus* in food [23]. The genes commonly used for bacterial typing were 16SrRNA [24], *tuf*, and *pheS* [25]. To determine the mean similarity with other LAB by Genbank, comparison analysis showed that the lowest similarity was 74.06% for *tuf*, 74.71% for *pheS*, and 89.79% for 16SrRNA. The lower the gene sequence similarity, the greater the interspecific distinction. This experimental solution fully demonstrated the high specificity and accuracy of using the *tuf* gene as a template for the qualitative and quantitative detection of six LABs. Jean-Pierre Furet et al. designed the primers using the 16SrRNA gene as a template to appear as multiple bands [26], whereas our primers based on the *tuf* gene were designed as single bands with high specificity. Therefore, the *tuf* gene was selected as our target gene.

In terms of LAB DNA extraction, the content of protein and fat in mixed fermented yogurt was high, and the yogurt was colloidal, which can effectively affect the yield of DNA extraction in yogurt. Therefore, when extracting LAB DNA from yogurt, the sample was diluted 15 times and mixed with proteinase K and SDS (8.0%). After that, high-speed centrifugation was performed to improve the yield of DNA and obtain high purity DNA [13]. Compared to only used TIANamp Bacteria DNA Kit, the DNA extracted in this study was higher, probably because proteinase K and SDS (8.0%) can effectively hydrolyze protein and fat and release the encapsulated LAB. Jean-Pierre Furet used a similar method in extracting LAB DNA from yogurt [26]. However, in this experiment, the DNA was extracted at higher yield and purity by using a 15-fold dilution followed by the addition of proteinase K and then concentration.

In the single-bacteria fermentation broth, the value obtained by the qPCR method was slightly higher than the plate count, which might be due to the plate count only counting the live bacteria, and there might be dead but undecomposed LAB DNA in the fermentation broth [27]. Although qPCR’s issue was not being able to distinguish between live and dead bacteria, the yogurt was generally fermented after 6 h, the LAB in this yogurt was in the logarithmic growth phase and had the strongest resistance to adverse environments; thus, the number of dead bacteria is relatively small [28]. This conclusion could also be drawn from the counting results of the two methods in the single bacteria fermentation broth at the same order of magnitude. On the whole, in the compound fermented yogurt, the result of using qPCR to count is slightly lower than the plate colony count, and the results are consistent with previous studies [25]. That is because when the selective counting plate was used to count each type of LAB in the yogurt, it could not be strictly distinguished. Thus, in the same counting plate, there were two or three types of LAB [28]. Except for the special morphology of *Lactobacillus acidophilus*, most of the six kinds of LAB were similar in morphology, and distinguishing them strictly with the naked eye on the plate was difficult, resulting in a large count. However, the overall count results were in the same order of magnitude, thus the qualitative and quantitative results of LAB in yogurt by qPCR were accurate and reliable.

According to literature reports, when the slope of the standard curve was between −3.1 and −3.6, and the correlation coefficient R^2^ was ≥0.99, the standard curve that meets the above conditions can be used for the quantitative analysis of strains [29]. Moreover, only a single melting peak appeared in the melting curve, and no non-specific dimers were produced. The specificity of the quantitative system was satisfactory, and the primers and PCR reaction conditions used were suitable for the qPCR reaction [30].

LIVE/DEAD BacLight Bacterial Viability Kits are a mixture of SYTO 9 green fluorescent nucleic acid dye and red fluorescent nucleic acid dye PI. SYTO 9 stain can usually label all bacteria in the population, whereas PI can only penetrate the cell membrane and damage the cell membrane. When both dyes are present, the fluorescence intensity of SYTO 9 decreases [31]. Therefore, using a mixture of SYTO 9 and PI stains, live bacteria with intact cell membranes will be stained green, and dead bacteria with damaged cell membranes will be stained red [32]. Therefore, the detection of LAB in yogurt using FCM combined with SYTO 9 and PI dye to distinguish the number of live and dead bacteria showed that the detection of total bacteria was similar to that obtained using the qPCR method, which proved that the qPCR count results were reliable. Although flow cytometry can distinguish between live and dead bacteria, it was not possible to detect LAB qualitatively in a multi-strain fermented sample, such as yogurt. In the laser confocal image, the green fluorescence points representing the live LAB in the yogurt are all above 90%, which was also consistent with the FCM assay results, indicating that the number of live bacteria in the yogurt after the fermentation was relatively high. Although yogurt contains about 10% dead LAB, according to the latest literature, some LAB such as *Lactobacillus acidophilus* [32], *Lactiplantibacillus plantarum* [33], and *Lacticaseibacillus rhamnosus* still maintain their original utility in an inactivated state [34], indicating that dead bacteria also had some probiotic function [35]. Therefore, the qPCR method used in this experiment was used to analyze the LAB in the yogurt qualitatively and quantitatively. The result was credible and closer to the true content of LAB in yogurt.

The qPCR method developed in this study based on the *tuf* gene allowed the qualitative and quantitative detection of six LAB in yogurt and the ability to count the distribution of live and dead bacteria of six LAB in yogurt. Since this method has the advantages of being timesaving, qualitative, and quantitative able to distinguish between live and dead bacteria, it might also be more in line with the product testing requirements of dairy enterprises, and this method might also have potential application in the detection of contaminating bacteria in fermented foods [36,37].

## 5. Conclusions

In this study, the *tuf* gene was used as the target DNA sequences to design specific primers, when after we compared the *tuf* gene with 16S rRNA gene by multiple alignments, and the copy number confirmed that *tuf* gene was more excellent than 16SrRNA. A novel qPCR method was established to detect *Streptococcus thermophilus*, *Lactobacillus delbrueckii subsp. bulgaricus*, *Lactobacillus acidophilus*, *Limosilactobacillus fermentum*, *Lacticaseibacillus casei*, and *Levilactobacillus brevis*. The results of laser confocal microscopy, traditional plate counting method, and FCM confirmed that the qPCR method was closer to the true level of LAB in yogurt, and the results were reliable. Compared with the existing methods, the method in this study allows the qualitative and quantitative detection of the six kinds of LAB in yogurt, and the distribution of live and dead bacteria of the six kinds of LAB in yogurt can be calculated. In subsequent experiments, a method based on this approach might be designed by us that could provide rapid detection of contaminating pathogenic bacteria in foods.

## Figures and Tables

**Table 1:** PCR reaction conditions.

PCR Amplification System (25 μL)	Volume (μL)
2× Taq PCR MasterMix	12.5 μL
DNA	1 μL (150 ng/μL)
Forward primers	1 μL (10 μmol/μL)
Reverse primers	1 μL (10 μmol/μL)
ddH_2_O	9.5 μL

**Table 2:** PCR reaction program.

PCR Reaction Program	Time		Cycle
94 °C	5 min		
94 °C	30 s	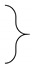	
58 °C	30 s		30
72 °C	30 s		
72 °C	10 min		
4 °C	save		

**Table 3:** Enzyme digestion system.

	Volume (μL)
pUC19	11.5 μL
10× Buffer	2 μL
SacI	1 μL
Hindlll	1 μL
ddH_2_O	4.5 μL

**Table 4:** The 16S rRNA gene copy number of 6 strains of lactic acid bacteria (LAB).

Data Source Record id	Data Source Organism Name	RDP Taxa	16S Copies	Genomes	Range	Median	Mean
GCF_003047065.1	*Lactobacillus acidophilus*	*Lactobacillus* (genus)	4	8	4–5	4	4.1
GCF_003952845.1	*Lactobacillus acidophilus*	*Lactobacillus* (genus)	5				
GCF_013342945.1	*Lactobacillus acidophilus*	*Lactobacillus* (genus)	4				
GCF_000389675.2	*Lactobacillus acidophilus La-14*	*Lactobacillus* (genus)	4				
GCF_000011985.1	*Lactobacillus acidophilus NCFM*	*Lactobacillus* (genus)	4				
GCF_001469775.1	*Lactobacillus delbrueckii* subsp. *bulgaricus*	*Lactobacillus* (genus)	8	12	8–9	9	8.8
GCF_011044195.1	*Lactobacillus delbrueckii* subsp. *bulgaricus*	*Lactobacillus* (genus)	8				
GCF_000191165.1	*Lactobacillus delbrueckii* subsp. *bulgaricus 2038*	*Lactobacillus* (genus)	9				
GCF_000056065.1	*Lactobacillus delbrueckii* subsp. *bulgaricus ATCC 11842 = JCM 1002*	*Lactobacillus* (genus)	9				
GCF_000014405.1	*Lactobacillus delbrueckii* subsp. *bulgaricus ATCC BAA-365*	*Lactobacillus* (genus)	9				
GCF_000466785.3	*Lactobacillus fermentum 3872*	*Lactobacillus* (genus)	5	20	1–5	5	4.8
GCF_000397165.1	*Lactobacillus fermentum F-6*	*Lactobacillus* (genus)	5				
GCF_000010145.1	*Lactobacillus fermentum IFO 3956*	*Lactobacillus* (genus)	5				
GCF_002192435.1	*Limosilactobacillus fermentum*	*Lactobacillus* (genus)	1				
GCF_002242615.1	*Limosilactobacillus fermentum*	*Lactobacillus* (genus)	5				
GCF_003255875.1	*Lactobacillus brevis*	*Lactobacillus* (genus)	4	18	4–6	5	5
GCF_003346795.1	*Lactobacillus brevis*	*Lactobacillus* (genus)	6				
GCF_001676805.1	*Lactobacillus brevis*	*Lactobacillus* (genus)	5				
GCF_006228245.1	*Lactobacillus brevis ATCC 367*	*Lactobacillus* (genus)	5				
GCF_006228265.1	*Lactobacillus brevis KB290*	*Lactobacillus* (genus)	5				
GCF_006228285.1	*Lactobacillus casei*	*Lactobacillus* (genus)	5	5	5–5	5	5
GCF_006228305.1	*Lactobacillus casei*	*Lactobacillus* (genus)	5				
GCF_014905055.1	*Lactobacillus casei 12A*	*Lactobacillus* (genus)	5				
GCF_900475625.1	*Lactobacillus casei DSM 20011 = JCM 1134 = ATCC 393*	*Lactobacillus* (genus)	5				
GCF_000014465.1	*Lactobacillus zeae*	*Lactobacillus* (genus)	5				
GCF_013307285.1	*Streptococcus thermophilus JIM 8232*	*Streptococcus* (genus)	6	66	5–6	6	5.7
GCF_015190465.1	*Streptococcus thermophilus LMD-9*	*Streptococcus* (genus)	6				
GCF_900094135.1	*Streptococcus thermophilus LMG 18311*	*Streptococcus* (genus)	6				
GCF_900474985.1	*Streptococcus thermophilus MN-ZLW-002*	*Streptococcus* (genus)	5				
GCF_000698885.1	*Streptococcus thermophilus ND03*	*Streptococcus* (genus)	5				

**Table 5:** Specific primers for six strains of lactic acid bacteria (LAB).

Strain	Forward Primer	Reverse Primers
LA	5′-GTGACAAGGAAGCTCAAGACCAA-3′	5′-CCACGACCAGTGATAGTGAATACG-3′
LB	5′-AAGCCATTCTTGATGCCAGTTGA-3′	5′-ACCAGTAACCGTCGTCTTCAGT-3′
LC	5′-TGAAGGCGACAAGGAACAGGAA-3′	5′-AAGCAACAGTACCACGACCAGT-3′
LD	5′-TGACGAATACATTCCAACTCCAGAAC-3′	5′-TCAACGCTGTCGCCAACCT-3′
LF	5′-GGAAGTCGTATTCGGACAGAAGGT-3′	5′-CTCGCCAGGTCGGTGTTGAA-3′
ST	5′-CGTGGTGTTGTTCGTGTTAATGA-3′	5′-CGGCAATACCTTCATCAAGTTGT-3′

**Table 6:** Homology arm primer.

Strain	Primer	Sequence
LA_2_	Sense	acggccagtgaattcgagctcATGGCAGAAAAAGAACATTACGTTAG
	Antisense	gaccatgattacgccaagcttTTAGTCAAGGATTTCAGTAACTTGACC
LB_2_	Sense	acggccagtgaattcgagctcATGGCTGAAAAAGAACATTATGAAAG
	Antisense	gaccatgattacgccaagcttTTAGTCGTCAATTTCCGTAACGG
LC_2_	Sense	acggccagtgaattcgagctcATGGCTGAAAAAGAACACTATGAACG
	Antisense	gaccatgattacgccaagcttTTAGTCAAGAATTTCGGAAACAACG
LD_2_	Sense	acggccagtgaattcgagctcATGGCAGAAAAAGAACATTACGTTAG
	Antisense	gaccatgattacgccaagcttTTAGTCGTCAATTTCAGTAACTTGGC
LF_2_	Sense	acggccagtgaattcgagctcTTAGTCGAGCACTTCGGATACCA
	Antisense	gaccatgattacgccaagcttATGGCAGAAAAAGAACATTATGAACG
ST_2_	Sense	acggccagtgaattcgagctcATGGCAAAAGAAAAATACGATCG
	Antisense	gaccatgattacgccaagcttTTAAGCTTCGATTTCAGATACGATACC

**Table 7:** Enumeration of LAB using FCM.

Type of Yogurt	Active Bacteria (lg CFU/mL)	Dead Bacteria (lg CFU/mL)
ST + LD	7.76 ± 0.12	6.85 ± 0.03
ST + LD + LA	7.94 ± 0.05	6.97 ± 0.11
ST + LD + LB	7.84 ± 0.04	6.94 ± 0.04
ST + LD + LC	7.99 ± 0.08	6.94 ± 0.06
ST + LD + LF	7.86 ± 0.05	6.92 ± 0.04

## Data Availability

The datasets generated for this study are available on request to the corresponding author.
